# Supplementary material for: Male emergence schedule and dispersal behaviour are modified by mate availability in heterogeneous landscapes: evidence from the orange-tip butterfly
Source: PeerJ. 2015 Jan 6;3:e707. doi: 10.7717/peerj.707 (PMC4304852; doi:10.7717/peerj.707)
Supplement: Appendix SI [file peerj-03-707-s001.docx]

For any population of regularly sampled mobile living organisms, the number of recaptures depends on two parameters: the residence-rate (describing the proportion of specimens retained in the population between sampling occasions) and the encounter-rate (describing the proportion of specimens resident in the population which are encountered on each sampling occasion). The residence-rate determines the average period (called the residence time) an individual remains in the population; it is directly proportional to the survival-rate and inversely proportional to the emigration-rate. The encounter-rate determines the average time (recapture period) between successive encounters (captures) of the same individual during its residency in the population. Intuitively, the longer the residence time, and the shorter the recapture period, the more individuals will be recaptured at least once.

The residence-rate can be estimated from MRR data by plotting the number of specimens remaining in the population against time. The encounter data for the whole season are synchronized by setting the day of first capture for all individuals to zero; a specimen is regarded as resident in the population every day until the day of last capture. If the decline is exponential (as is usual for insect populations), then a logarithmic transformation will yield a straight line graph (here termed the residence plot). The gradient (*m*) of this line gives the residence-rate as exp(*m*) and the residence time as -1/*m* (where the minus sign corrects for the negative value of *m*). The encounter-rate can be found in a similar way by plotting the number of recaptures remaining against time, in which all recaptures are synchronized by setting the date of previous release to zero. For example, a specimen recaptured 1, 4, 8 and 10 days after initial capture would contribute four recaptures to the total number, which after synchronization would occur after 1, 3, 4, and 2 days. Provided the decay is exponential, a logarithmic transformation will produce a straight line graph (the recapture plot), whose gradient *(g*) yields the encounter-rate as [1 - exp(*g*)] (since exp(*g*) gives the rate at which specimens *avoid* recapture) and the recapture period as -1/*g*.

Since insect populations are usually sampled on a daily basis, the gradients *m* and *g* express the logarithmic decline in the number of specimens/recaptures *per day*; hence, the *daily* residence-rate and the *daily* encounter-rate are calculated from them. It is possible to express these parameters in alternative time units by modifying their formulae to exp(*mt*) and [1 - exp(*gt*)] respectively, where *t* indicates the desired time unit in days. For example, the residence-rate of specimens over half-day and two-day intervals is given by exp(*m*/2) and exp(2*m*) respectively.

Having obtained *m* and *g* from the residence and recapture plots, the fraction of specimens predicted to be recaptured at least once can be calculated. We first *exclude* recaptures obtained on the day of initial capture, since it is very easy to recapture a specimen within minutes of its release, and therefore very difficult to decide on a convention as to which recaptures to include as 'genuine'. Hence, we confine our attention to recaptures made from the day after release (day 1) onwards.

Let

*N* = total number of specimens captured (taken to occur on day 0).

*n* = actual number of specimens in the population on day 1.

*n'* = known number of specimens in the population on day 1, equal to the total number of specimens eventually recaptured.

*F* = fraction of specimens recaptured (= *n'*/*N*).

*s* = daily residence-rate (= $e^{m}$).

*p* = daily encounter-rate (= $1-e^{g}$).

If *N* specimens are caught on day 0 then the number remaining on day 1, *n*, will be

$n=Ne^{m/2}$

where we have assumed that specimens are (on average) first encountered half way through day 0, so that the proportion remaining on day 1 is found by substituting *t* = 1/2 into exp(*mt*). Of these

$np=e^{m/2}Np$

are actually captured.

Therefore $e^{m/2}Np$ provides the first contribution to the total number of specimens recaptured (*n'*). Having made this contribution, these specimens are now redundant and in order to avoid counting them twice they must be subtracted from *n*, to give the number of unrecorded specimens remaining after day 1 as

$e^{m/2}N(1-p)$

To obtain the number of unrecorded specimens remaining on day 2, this is multiplied by *s*; to obtain the number caught on day 2, the result in turn is multiplied by *p*; therefore the second contribution to *n'* is

$e^{m/2}N(1-p)sp=\left(e^{m/2}Np\right)\left[\left(1-p\right)s\right]$

Repeating this process, the third contribution to *n'* is

([Number of specimens present on day 2] – [Number of specimens caught on day 2]) x *s* x *p*

$$=\left(e^{m/2}N\left(1-p\right)s-e^{m/2}N\left(1-p\right)sp\right)sp$$

$$=\left(e^{m/2}N\left(1-p\right)s\right)\left(1-p\right)sp=\left(e^{m/2}Np\right)\left(1-p\right)^{2}s^{2}$$

$$=\left(e^{m/2}Np\right)\left[\left(1-p\right)s\right]^{2}$$

Inspection of the first three contributions to the total number of specimens recaptured, viz $e^{m/2}Np$, $\left(e^{m/2}Np\right)\left[\left(1-p\right)s\right]$, and $\left(e^{m/2}Np\right)\left[\left(1-p\right)s\right]^{2}$indicates that they form the first three terms of a Geometric Progression, with first term $e^{m/2}Np$and common ratio$\left(1-p\right)s$. Therefore summing the terms to infinity

$$n'=\frac{e^{m/2}Np}{1-\left(1-p\right)s}$$

and the total fraction of specimens recaptured is

$$F=\frac{e^{m/2}p}{1-\left(1-p\right)s}$$

This equation gives the predicted value of *F* in terms of the daily residence-rate (*s*) and the daily encounter-rate (*p*). Substituting $s=e^{m}$ and $p=1-e^{g}$

$$F=\frac{e^{m/2}\left(1-e^{g}\right)}{1-e^{g}e^{m}}=\frac{e^{m/2}\left(1-e^{g}\right)}{1-e^{g+m}}$$

As it stands, this equation is not accurate since it was derived from a Geometrical Progression representing the estimated number of recaptures obtained at discrete daily intervals; since sampling effort is continuous, this provides only a crude representation of the recapture process. Therefore, the correct formula for *F* will only be found by introducing *t* into the equations for *s* and *p* and letting it tend to zero (which effectively converts recapture into a continuous process). Hence

$$F=\frac{e^{m/2}\left(1-e^{gt}\right)}{1-e^{\left(g+m\right)t}}$$

which is insoluble as *t* → 0, being of the form 0/0. It is therefore necessary to employ l’Hôpital’s rule and differentiate both numerator and denominator, giving

$$\frac{-e^{m/2}ge^{gt}}{-\left(g+m\right)e^{\left(g+m\right)t}}$$

which, on letting *t* tend to zero, gives

$$F=e^{m/2}\frac{g}{g+m}$$

as the correct formula for the fraction of specimens predicted to be recaptured at least once.

An intuitive grasp of this equation can be gained by considering the effects of extreme values of *m* and *g*. Thus, as $m\to0$, death and emigration will cease and so all specimens will be recaptured eventually ($F\to1$); as $m\to-\infty$, death and emigration will be instantaneous and so no specimens will be recaptured ($F\to0$). On the other hand, if $g\to0$, encounters will cease and so no specimens will be recaptured ($F\to0$); whereas if $g\to-\infty$, specimens will be encountered immediately and so all available on day 1 will be recaptured ($F\to e^{m/2}$).

As a bonus, we note that an estimate of the size of the population (*N_pop_*) being sampled can be obtained from 1/*F* (where the term $e^{m/2}$is dropped since we do not have to wait an initial period before the specimens become available for capture). Hence

$$N_{pop}=\frac{g+m}{g}$$

It should be noted, however, that this equation is dependent on the assumption of uniform behaviour; if there is a 'stay-or-go' response, such that some specimens emigrate from the population immediately, the estimate obtained will not be accurate.
