# Supplementary material for: Male emergence schedule and dispersal behaviour are modified by mate availability in heterogeneous landscapes: evidence from the orange-tip butterfly
Source: PeerJ. 2015 Jan 6;3:e707. doi: 10.7717/peerj.707 (PMC4304852; doi:10.7717/peerj.707)
Supplement: Appendix SII [file peerj-03-707-s002.docx]

In order to see how source-sink structure might impact on the evolution of emergence timing through dispersal, we make three key assumptions (our analysis owes a debt to the derivation of Pulliam (1988), but our assumptions are very different).

1. Since we are interested in males only, we equate reproductive success with the number of matings obtained, instead of the more direct measure of the number of offspring produced (this implicitly assumes that all matings are of equal value to males). Since females are monandrous, the number of mating opportunities for males is equal to the number of females. We take the sex-ratio to be equal.

2. In heterogeneous landscapes, we assume two basic types of habitat, determined by population density: high density *sources*, in which emerging males encounter (and mate with) all females, and low density *sinks*, in which emerging males do not encounter all females. (For simplicity, we hereafter consider a single source and sink.)

3. Within the source population there will be dominants and subordinates; that is, some males (dominants) achieve >1 matings and others (subordinates) <1. We assume that subordinates improve their fitness by emigrating to the sink, although their fitness remains <1 (they are effectively "making the best of a bad job"). In our model, dominance relationships in the source are determined by emergence timing, and subordinates increase their fitness in the sink due to the lower population density (less intense mate competition) there. We assume a simple response in which males emerging in the source either 'stay' (dominants) or 'go' to the sink immediately (subordinates).

Let

*n_s_* = number of 'stay' males emerging in source.

*n_g_* = number of 'go' males emerging in source.

*n_m_* = total number of males emerging in source (= *n_s_ + n_g_*).

*n_f_* = number of females emerging in source (= *n_m_* for an equal sex-ratio).

*n* = number of males plus females emerging in the source; for an equal sex-ratio *n*/2 = *n_f_* = *n_m_*.

*λs* = average number of matings obtained by 'stay' males in source; λs > 1 by assumption 3.

*λg* = average number of matings obtained by males in sink (whether emerging in source ('go' males) or sink); λg < 1 by assumption 3.

*n_skm_/n_skf_* = total population of males/females *emerging* in sink (for males, before immigration of "go" specimens).

*n_sk_* = number of males plus females emerging in the sink; for an equal sex-ratio *n_sk_*/2 = *n_skm_* = *n_skf_*.

*N* = number of males plus females in the whole population (source + sink).

α = proportion of females mated in the sink. (In the source, all females are mated by assumption 2.)

We shall first derive equations giving the number of specimens of each type emerging in the landscape, together with ones describing their reproductive success. Since 'stay' males mate with all the source females:

$n_{f}=n_{s}\lambda s$ so $\lambda s=\frac{n_{f}}{n_{s}}=\frac{n}{2n_{s}}$ (I)

or $n_{s}=\frac{n}{2\lambda s}$ (II)

on the assumption of an equal sex-ratio (*n_f_* = *n_m_* = *n*/2). So

$$n_{g}=n_{m}-n_{s}=\frac{n}{2}-n_{s}$$

$n_{g}=\frac{n}{2}\left(1-\frac{1}{\lambda s}\right)$ (III)

Since sink females are mated either by males emerging in the sink or by 'go' males emerging in the source:

$$n_{skm}\lambda g+n_{g}\lambda g=\alpha n_{skf}$$

$\lambda g=\frac{\alpha\frac{n_{sk}}{2}}{\frac{n_{sk}}{2}+n_{g}}$ (IV)

$$\frac{n_{sk}}{2}\left(\lambda g-\alpha\right)=-n_{g}\lambda g$$

$n_{sk}=\frac{2n_{g}\lambda g}{\left(\alpha-\lambda g\right)}$ (Va)

$n_{sk}=\frac{n\left(1-\frac{1}{\lambda s}\right)\lambda g}{\alpha-\lambda g}$ (Vb)

Note from equation (IV) that *λg* < *α*, so the denominator in (Vb) is positive. The total number of specimens is:

$$N=2\left(n_{s}+n_{g}\right)+n_{sk}$$

$$=\frac{n}{\lambda s}+\left(n-\frac{n}{\lambda s}\right)+\frac{n\left(1-\frac{1}{\lambda s}\right)\lambda g}{\alpha-\lambda g}$$

$$=\frac{n\left(\alpha-\lambda g\right)+n\left(\lambda g-\frac{\lambda g}{\lambda s}\right)}{\alpha-\lambda g}$$

$N=\frac{n\left(\alpha-\frac{\lambda g}{\lambda s}\right)}{\alpha-\lambda g}$ (VI)

These equations give the numbers and fitnesses of specimens emerging in different parts of the landscape, but they neglect the cause of the 'stay-or-go' response, i.e. the assumption that source 'go' males improve their fitness in the sink (assumption 3). If we assume that the *predicted* fitness in the source (*before* the emigration of 'go' specimens) is normally distributed (for justification see below), then we can express the proportion of males whose predicted source fitness falls below the average sink fitness in terms of the cumulative probability function of the normal distribution. This is expressed in terms of the standard variable *Z*, such that the probability that *Z* lies below some standardized value of interest, *z*, is

$$\Phi\left(z\right)=\Pr(Z<z)$$

$$z=\frac{x-\mu}{\sigma}$$

where *µ* is the mean, *σ* the standard deviation and *x* the actual value of interest. In our case, we are interested in the fraction of males in the source (where the mean predicted fitness is 1 by assumption 2: since males emerging in the source mate with all the source females, a 1:1 sex-ratio (assumption 1) guarantees that on average each male gains 1 mating) with predicted fitness below *λg*; so

$$z=\frac{\lambda g-1}{\sigma}$$

where *σ* is the standard deviation in the predicted fitness of males emerging in the source. Hence, $\Phi\left(\left(\lambda g-1\right)/\sigma\right)$will give the probability that source males will have a predicted fitness below *λg*; assuming that these emigrate to the sink, we can rewrite the number of 'stay' and 'go' males as

$n_{s}=n_{m}\left(1-\Phi\left(\frac{\lambda g-1}{\sigma}\right)\right)=\frac{n}{2}\left(1-\Phi\left(\frac{\lambda g-1}{\sigma}\right)\right)$ (VII)

$n_{g}=n_{m}\Phi\left(\frac{\lambda g-1}{\sigma}\right)=\frac{n}{2}\Phi\left(\frac{\lambda g-1}{\sigma}\right)$ (VIII)

Substituting (VIII) in (Va), the total number of males and females emerging in the sink is

$n_{sk}=\frac{n\left(\Phi\left(\frac{\lambda g-1}{\sigma}\right)\right)\lambda g}{\alpha-\lambda g}$ (IX)

which can be rearranged to give

$\Phi\left(\frac{\lambda g-1}{\sigma}\right)=\frac{n_{sk}\left(\alpha-\lambda g\right)}{n\lambda g}$ (X)

If we now assume that the variance in predicted source male fitness (= *σ*^2^) is caused by departures from an ESS distribution of emergence times, this equation expresses the relationship between precision in male emergence timing and the size of a sink population in heterogeneous landscapes. Since *λg* < 1, (*λg* - 1)/*σ* is negative and therefore Φ increases with increasing *σ*; hence, the sink population is boosted by increasing *σ* or decreasing precision in emergence timing. Conversely, a large sink population could maintain high *σ* and hence relieve selection pressure for precise emergence timing; or, if such precision is beyond the evolutionary reach of the organism, the existence of a sink could allow maladaptively emerging males to boost their fitness in a way that would otherwise be unavailable to them.

It is important to be clear that the variance in predicted fitness described by *σ*^2^ refers *only* to that caused by departures from an ESS emergence schedule (i.e. by imperfect emergence timing); if the emergence curve is an ESS (perfect emergence timing), then *all* males are predicted to obtain 1 mating and hence *σ*^2^ = 0. That is, the additional variance due to sampling error is not included in *σ*^2^. (In the field, mating success should be Poisson distributed, so we would anticipate that departures from an ESS would result in intra-seasonal changes in the mean/variance of this distribution; incorporation of this effect is beyond the scope of the current analysis.) Our assumption that imperfect emergence timing leads to a normal distribution in predicted fitness values over the course of the whole season can be tested in our study population by plotting the frequency of the values obtained from equation 3 over the six-year study period. The result is a reasonable approximation to a Gaussian distribution with *μ* = 1 and *σ* = 0.46 (Fig. A1), so this assumption may be accepted.

We have assumed that some females in the sink remain unmated (*α* < 1). This is in line with known Allee effects in many organisms (decreasing population growth with decreasing population size due to low mate encounter rates). If, however, an equilibrium is reached in which emigrating males from the source, coupled with those emerging in the sink, mate with all sink females, then we may set *α* = 1 in the above equations (as we have done in the main text). In this case, a 'stay-or-go' response resulting from imprecise emergence timing in the source would be responsible for maintaining a stable population in the sink, which would otherwise require repeated repopulation through emigrating source females to avoid extinction through Allee effects.
